# 

**Published:** 1867-04

**Authors:** 


Vol. VIII. **APRIL, 1867.** No. 2.

### ATLANTA
### Medical and Surgical
### JOURNAL.

NEW SERIES.

EDITED BY

**J. G. WESTMORELAND, M. D.,**
*Professor of Materia Medica and Therapeutics in the Atlanta Medical College.*

**W. F. WESTMORELAND, M. D.,**
*Professor of the Principles and Practice of Surgery in the Atlanta Medical College.*

AND

**J. M. JOHNSON, M. D.**

**Pax et scientia, sed veritas sine timore.**

**PUBLISHED MONTHLY AT $4.00 PER ANNUM, IN ADVANCE**

ATLANTA, GA.:
PRINTED AT THE ATLANTA INTELLIGENCER BOOK & JOB OFFICE.
1867.

Postage 36 cts. a year, pre-paid quarterly. Postmasters are requested to compl with the law, bv giving us notice when the Journal is refused at their Offices.

